# Voltammetric study of cefotaxime at the macroscopic and miniaturized interface between two immiscible electrolyte solutions

**DOI:** 10.1007/s00604-021-05072-w

**Published:** 2021-11-09

**Authors:** Konrad Rudnicki, Karolina Sobczak, Magdalena Kaliszczak, Karolina Sipa, Emilia Powałka, Sławomira Skrzypek, Lukasz Poltorak, Gregoire Herzog

**Affiliations:** 1 Department of Inorganic and Analytical Chemistry, Faculty of Chemistry, University of Lodz, Tamka 12, 91-403 Lodz, Poland; 2 Université de Lorraine, CNRS, LCPME, Nancy, France

**Keywords:** ITIES, Electrified liquid-liquid interface, Fused silica capillaries, Cefotaxime, AC voltammetry

## Abstract

The electrochemical behavior of cefotaxime (CTX^+^) was investigated at the polarized macro- and micro-interface between two immiscible electrolyte solutions (ITIES) by cyclic voltammetry and alternating current voltammetry. Miniaturization was achieved with fused silica microcapillary tubing entrapped in a polymeric casing. Scanning electron microscopy (SEM) was employed for the fabricated LLI support characterization. Voltammetric investigation of CTX^+^ at macro- and μ-ITIES allowed the determination of many physicochemical parameters, such as formal Galvani potential of the ion transfer reaction (${\Delta }_{org}^{aq}{\varPhi}^{\prime }$), diffusion coefficients (*D*), formal free Gibbs energy of the ion transfer reaction (∆*G*^′*aq* → *org*^), and water-1,2-dichloroethane partition coefficient (${\log}{P}_{water/ DCE}^{CTX+}$). Additionally, based on the results obtained the analytical parameters including voltammetric sensitivity, limits of detection and the limits of quantification (in micromolar range) were calculated. The applicability of the developed procedures was verified in spiked still mineral and tap water samples.

**Graphical abstract:**

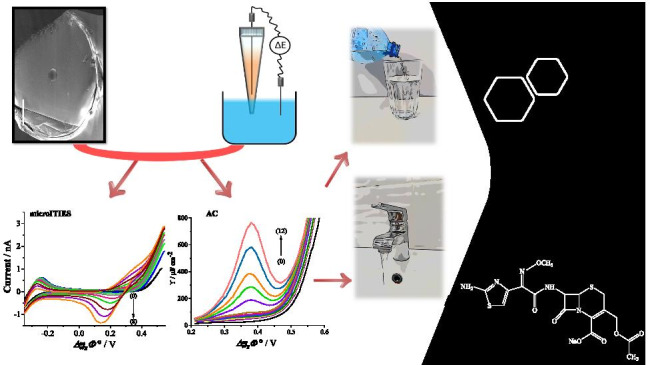

**Supplementary Information:**

The online version contains supplementary material available at 10.1007/s00604-021-05072-w.

## Introduction

**Fig. 1.**
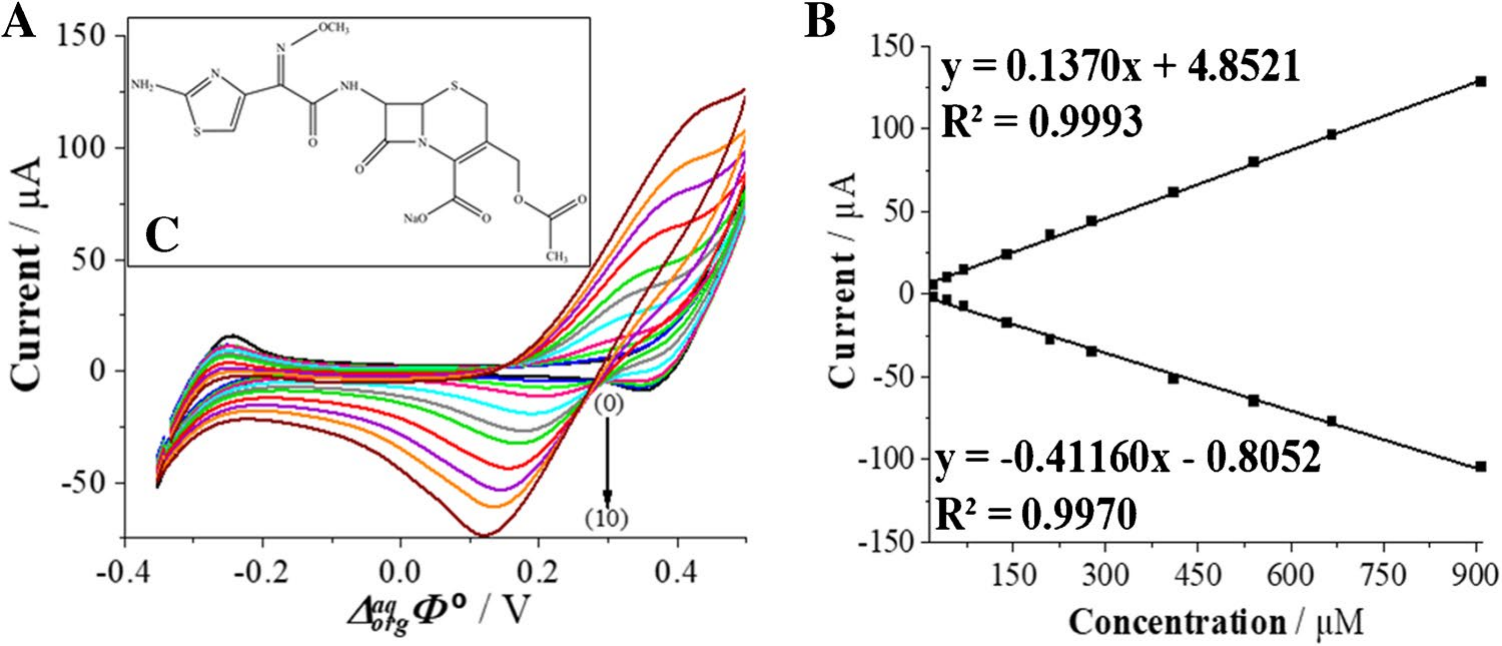
**A** Cyclic voltammograms (CVs) recorded for blank (0) and increasing CTX^+^ concentration (1–10) equal to 21.4; 42.7; 70.9; 140.8; 209.8; 277.8; 411.1; 540.5; 666.7 and 909.1 μM. The aqueous phase was 10 mM HCl (pH 2). **B** The corresponding forward (positive) and backward (negative) peak current intensities plotted in function of the CTX^+^ concentration. **C** The inset presents the structure of CTX^+^. Conditions: *v* = 20 mV s^−1^.

Cephalosporins (CFS) are semi-synthetic antibiotics of the β-lactam family. Their mechanism of action is based on the reduction of bacterial peptidoglycans consequently blocking their growth [1]. These antibiotics have a broad spectrum of activity against Gram-positive and Gram-negative aerobic and anaerobic bacteria. Cefotaxime (CTX, Fig. 1C) is the most active among third-generation CFS, particularly against Gram-negative bacteria as compared with the 1st and 2nd CFS generations [2]. In humans, these antibiotics are often used to treat urinary and respiratory tract bacterial infections. Preventive treatment of bacterial infections in cattle with CFS is a direct threat of their residues in food, especially meat and dairy products [3]. Antimicrobial resistance of bacteria together with the potential allergic reactions caused by CTX^+^ along with other members of β-lactam family are certainly a societal problem. In recent years, the consumption of antibiotics has increased dramatically leading their existence in natural environment. Especially due to this reasons, these chemical species must be monitored in the surrounding environments, human and animal biological fluids, or foodstuffs [2, 4].

A number of methods have been reported to date for the determination of CTX^+^. Examples include high-performance liquid chromatography (HPLC) [5, 6], liquid chromatography (LC) [7], chemiluminescence sensing [8], and also electroanalytical methods [4, 9, 10]. Low cost, simple instrumentation, existence of relatively easy miniaturization protocols, and integrability into electronic circuits speak for electrochemical techniques which are especially attractive from the presumptive sensing point of view. Electroanalytical sensing at solid electrodes can reach high or even very high selectivity usually for the cost of the elaborated surface engineering [11]. Detection of different chemical species, such as CTX^+^, at the solid electrodes, can be obstructed by the redox active interferences co-existing in real samples. (e.g., phenols passivating carbon-based electrodes are inactive at the ITIES when deprived from other ionizable functionalities) [12].

Electrochemistry at the interface between two immiscible electrolyte solutions (ITIES) provides an alternative to a conventional sensing at the solid electrified junctions [13]. Detection at ITIES is not limited to oxidation/reduction reactions; its mechanism is frequently related to ionic species transferring across the polarizable liquid-liquid interface (LLI). It is often used to detect charged molecules that are electrochemically inactive at conventional solid electrodes [14]. Moreover, since the ionic partitioning governed by the molecular lipophilicity is directly related to the Galvani potential difference of the ion transfer reaction, the signals originating from hydrophilic-hydrophobic analytes can be separated which in turn may improve detection selectivity. For all these reasons, ITIES was used to study many classes of chemical species, e.g., proteins [15], antibiotics [16], drugs [17], or polyelectrolytes [18].

Within last two decades, significant attention was given to the development of a variety of ITIES miniaturization protocols [19]. LLI downscaling brings a few benefits to electroanalytical studies. First of all, under proper geometrical conditions, the mass transfer of the analyte towards the microscopic junction is improved as compared with the macroscopic systems. This in turn improves the detection sensitivity. Capacitive currents drop with the ITIES surface area further lowering the voltammetric limits of detection. Small internal volumes of the miniaturized ITIES platforms drastically reduce amount of consumed reagents, especially toxic organic phase [16]. Finally, the properties of the miniaturized ITIES can be tuned by the surface chemistry of the supports. Lisiqi Xie et al. constructed nanoITIES formed within an ultrathin (80 nm thickness) isoporous silica membrane patterned with channels having 2–3 nm as the inner diameter and showed that the interfacial transfer of model ions was affected by the membrane surface charge controlled by the aqueous phase pH [20]. Size and charge sieving at the ITIES can be also achieved with silica deposits [21] or charged polymeric cushions [22]. The examples of other platforms used as an ITIES support include laser ablated pore(s) formed within polymeric [23] films or glass sheets [15], ion beam patterning of silicon nitride wafers [24], etched metal microwire electrodes formed in a glass casing [25], or pulled glass capillaries [26]. All these examples require the access to fabrication facilities and are burthen with rather timely preparation procedures. We have recently proposed a very fast μITIES fabrication protocol which uses fused silica micro-capillaries having 25 μm as an inner diameter. We have found that these platforms can be successfully used for electroanalytical applications, e.g., fluoroquinolone antibiotics screening [16, 27], food quality control [14], or as a support to electrochemically facilitated formation of nylon-6,6 at the ITIES [28].

In this work, we have miniaturized the ITIES formed at the water || 1,2-dichloroethane solutions to study interfacial behavior of one of the CFSs antibiotics — cefotaxime. Cyclic voltammetry (CV) allowed the determination of a number of electroanalytical and physicochemical parameters, e.g., formal Galvani potential of the ion transfer reaction (${\Delta }_{org}^{aq}{\varPhi}^{\prime }$), diffusion coefficients (*D*), formal free Gibbs energy of the ion transfer reaction (∆*G*^′*aq* → *org*^), and water-1,2-dichloroethane partition coefficient (${\log}{P}_{water/ DCE}^{CTX+}$). We also applied alternating current voltammetry (ACV) to study CTX^+^ in model and spiked real mineral water and tap water. The obtained results were validated and analytical parameters, this is limits of detection (LOD), limits of quantification (LOQ), linear dynamic range (LDR), and detection sensitivities were tabulated.

## Methods and materials

### Apparatus

**Scheme 1.**
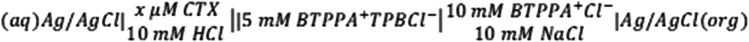
The composition of the cell used to study the electrochemical behavior of CTX^+^ at the macroITIES.

All electroanalytical measurements were carried out with the AUTOLAB–PGSTAT302N) equipped with the ECD module (Metrohm Autolab B.V., the Netherlands) controlled via NOVA 1.11.1 software. MacroITIES studies were performed in a four-electrode electrochemical system equipped with two platinum (Pt) wires acting as the counter electrodes and two silver/silver chloride wires (Ag/AgCl) applied as the reference electrodes (Scheme 1). The formal Galvani potential of the ion transfer was calculated using data from cyclic voltammograms with the potential axis calibrated using standard Galvani potential of tetrapropylammonium cation (${\Delta }_{org}^{aq}{\varPhi}_{TPrA^{+}}^0=-0.091\ V$) [29].

$$\left.(aq) Ag/ AgCl\right|{\displaystyle \begin{array}{c}x\ \mu M\ CTX\\ {}10\ mM\ HCl\ \end{array}}\left|\left|5\ mM\ {BTPPA}^{+}{TPBCl}^{-}\right.\right|{\displaystyle \begin{array}{c}10\ mM\ {BTPPA}^{+}{Cl}^{-}\\ {}10\ mM\ NaCl\end{array}}\left| Ag/ AgCl\right.(org)$$

**Scheme 2.**
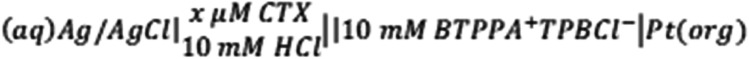
Electrochemical cell employed in μITIES studies.

The set-up used for μITIES polarization contained three electrodes, two in the aqueous phase and one in the organic phase. In the aqueous phase, the platinum wire (Pt) and silver/silver chloride wire (Ag/AgCl) were employed as the counter and reference electrode, respectively. In the organic phase, the platinum wire (Pt) was used as the auxiliary and the pseudo-reference electrode (Scheme 2). The μITIES system was constructed using a small piece of methyl deactivated (internal walls of the tubing are commercially functionalized with methyl groups) fused silica microcapillary tubing (FSMT) with inner diameter = 25 μm. The fabrication protocol of μ-platforms preparation is described elsewhere [27].

$$\left.(aq) Ag/ AgCl\right|{\displaystyle \begin{array}{c}x\ \mu M\ CTX\\ {}10\ mM\ HCl\end{array}}\left|\left|10\ mM\ {BTPPA}^{+}{TPBCl}^{-}\right.\right| Pt(org)$$

AC voltammetry measurements were performed under the following experimental conditions: frequency (*f*) 1 Hz, the amplitude (*E*) = 10 mV and the step potential (Δ*E*) = 10 mV. It was assumed that the μITIES system behaves as a Randles circuit. The analytical signal (ϒ) applied to plot the AC voltammograms was obtained according to Eq. (1) [30]:

1 $$\Upsilon =-\frac{10^6}{\upomega {Z}_{Im}{a}}-{C}_d$$

where *ω*, the angular frequency (rad·s^−1^); *Z*_*Im*_, the imaginary part of the impedance; *a*, the surface of the polarized LLI (*a* = 1.39 cm^2^); and *C*_*d*_, the double layer capacitance of the polarized LLI.

All measurements were conducted at room temperature (21.0 ± 2.0 °C).

The characterization of the FSMT used to fabricate μITIES platforms was performed with a scanning electron microscope (SEM, JCM-6000 Versatile Benchtop, JEOL, Japan). The beam energy of 5 kV was applied.

### Reagents and solutions

All solutions used in this work were prepared from analytical grade chemicals. Analytical standard of CTX^+^ (CAS No. 64485-93-4) was purchased from Sigma-Aldrich (France). CTX^+^ fresh stock solution (10 mM) was freshly prepared in 10 mL glass graduated flask by dissolving an appropriate amount of analyte in 10 mM hydrochloric acid (HCl) solution. Potassium tetrakis(4-chlorophenyl)borate (KTPBCl, > 98%) and bis(triphenylphosphoranylidene) ammonium chloride (BTPPACl, 97%) were also purchased from Sigma-Aldrich (France) and were used as the substrates for a bis(triphenylphosphoranylidene)ammonium tetrakis(4-chlorophenyl)borate (BTPPATPBCl) synthesis. Self-made BTPPATPBCl was dissolved in 1,2-dichloroethane (1,2-DCE, 99% Sigma-Aldrich, France) and was subsequently used as the supporting electrolyte for the organic phase. The tetramethylammonium chloride (TMACl, 97%, Sigma-Aldrich, France) was employed as a reference and model species. All aqueous solutions were prepared using deionized water, filtered using 0.22-μm filters (Millipore) and refrigerated at ± 4.0 °C. The FSMT with an internal diameter of 25 μm was obtained from VWR (Poland).

## Results and discussion

### macroITIES studies

At first, electrochemical behavior of CTX (the inset — Fig 1A) was studied at the macroITIES with CV and ACV. Chosen CVs recorded at macroITIES for increasing concentrations of CTX^+^ (always initially present in the aqueous phase) are shown in Fig. 1A. When dissolved in 10 mM HCl solution (pH set to 2) CTX is almost fully protonated (p*K*_*a1*_ = 3.42) [31]. Based on its structure inspection (inset of Fig. 1C), we have concluded that at given pH, CTX should exist mostly in a cationic form (concentration fraction of cationic form ~ 96%, see Fig. S1) with a delocalized positive charge located in between primary amine and nitrogen atom from the aminothiazole heterocycle. The carboxylate anions located on the opposite side of the CTX structure will be neutral. As such, positively charged CTX (CTX^+^) molecules may undergo the interfacial transport between the aqueous and the organic phases. During voltammetric studies the LLI was polarized from less to more positive potentials on the forward scan giving positive currents being related to the CTX^+^ transfer from the aqueous to the organic phase. As can be seen in Fig. 1A, the observed process was reversible with the forward and reversed current being close to unity and the peak-to-peak separation (Δ*E*_*p*_) extrapolated to 56 mV (see the intercept of Fig. S2 showing the increasing peak-to-peak separation plotted in function of the CTX^+^ concentration). The latter value is close to the theoretical 59 mV·z^−1^ (*z* = 1), and it points out that the CTX molecule is mono-charged. From the slope of the linear dependency of the signal current (*I*_*s*_) plotted against voltammetric scan rate (*v*) (Fig. S3) and the Randles-Ševčík equation, we have calculated the aqueous and the organic diffusion coefficients (*D*). Obtained values were equal to *D*_*aq→org*_ = 2.55 × 10^−6^ cm^2^·s^−1^ and *D*_*org→aq*_ = 4.62 × 10^−7^ cm^2^·s^−1^. Another parameter that can be determined from CVs is the formal Galvani potential of the ion transfer (${\Delta }_{org}^{aq}{\varPhi}^{\prime }$). This parameter is closely related to the hydrophobicity/hydrophilicity of studied molecule. For cationic species, the higher value of the ${\Delta }_{org}^{aq}{\varPhi}^{\prime }$ indicate their greater hydrophilicity [16]. The ${\Delta }_{org}^{aq}{\varPhi}^{\prime }$ can be further used (Eq. 2) to calculate the formal water – 1,2-DCE partition coefficient (${\log}{P}_{water/ DCE}^{CTX+}$) [32]:

2 $${\log}{P}_{water/ DCE}^{CTX+}=-\frac{\Delta _{org}^{aq}{\varPhi}^{\prime }{z}_iF}{2.303 RT}$$

where ${\Delta }_{org}^{aq}{\varPhi}^{\prime }$, the formal Galvani potential of the ion transfer reaction (in V); *z*_*i*_, charge of the studied molecule; *F*, the Faraday constant (96485 C·mol^−1^); *R*, the gas constant (8.314 J mol^−1^·K^−1^); and *T*, the temperature (298 K). The calculated ${\log}{P}_{water/ DCE}^{CTX+}$ for CTX^+^ is equal to − 4.48 which points out high hydrophilicity of the analyte, while based on the ${\log}{P}_{water/ octanol}^{CTX}$ = 0.64 available in the veterinary substances database [33], it can be stated that CTX is a weakly hydrophobic compound. Finally, the ${\Delta }_{org}^{aq}{\varPhi}^{\prime }$ was used to determine the formal Gibbs free energy of the ion transfer interfacial reaction (∆*G*^′, *aq* → *org*^), according to Eq. 3:

3 $$\Delta {G}^{\prime, aq\to org}={z}_iF{\Delta }_{org}^{aq}{\varPhi}^{\prime }$$

All physicochemical and electroanalytical parameters determined for CTX^+^ are collected and summarized in Table 1.

**Table 1.** Selected physicochemical and electroanalytical parameters for CTX^+^.

Analyte	z	*pKa*_1_	*pKa*_2_	D [cm^2^ s^−1^]_aq→org_^a^	D [cm^2^ s^−1^] _org→aq_^a^	${\log}{P}_{water/ octanol}^{CTX}$	${\log}{P}_{water/ DCE}^{CTX+}$^b^	${\Delta }_{org}^{aq}{\varPhi}^{\prime }$ [mV]	∆*G*^′, *aq* → *org*^ [kJ mol^−1^] ^c^
*CTX*	1	3.42 [31]	6.84[31]	2.55×10^-6^	4.62×10^-7^	0.64 [33]	-4.48	265	25.57

^a^Calculated from the Randles-Ševčík equation [14].

^b^See Eq. 2

^c^See Eq. 3

**Fig. 2.**
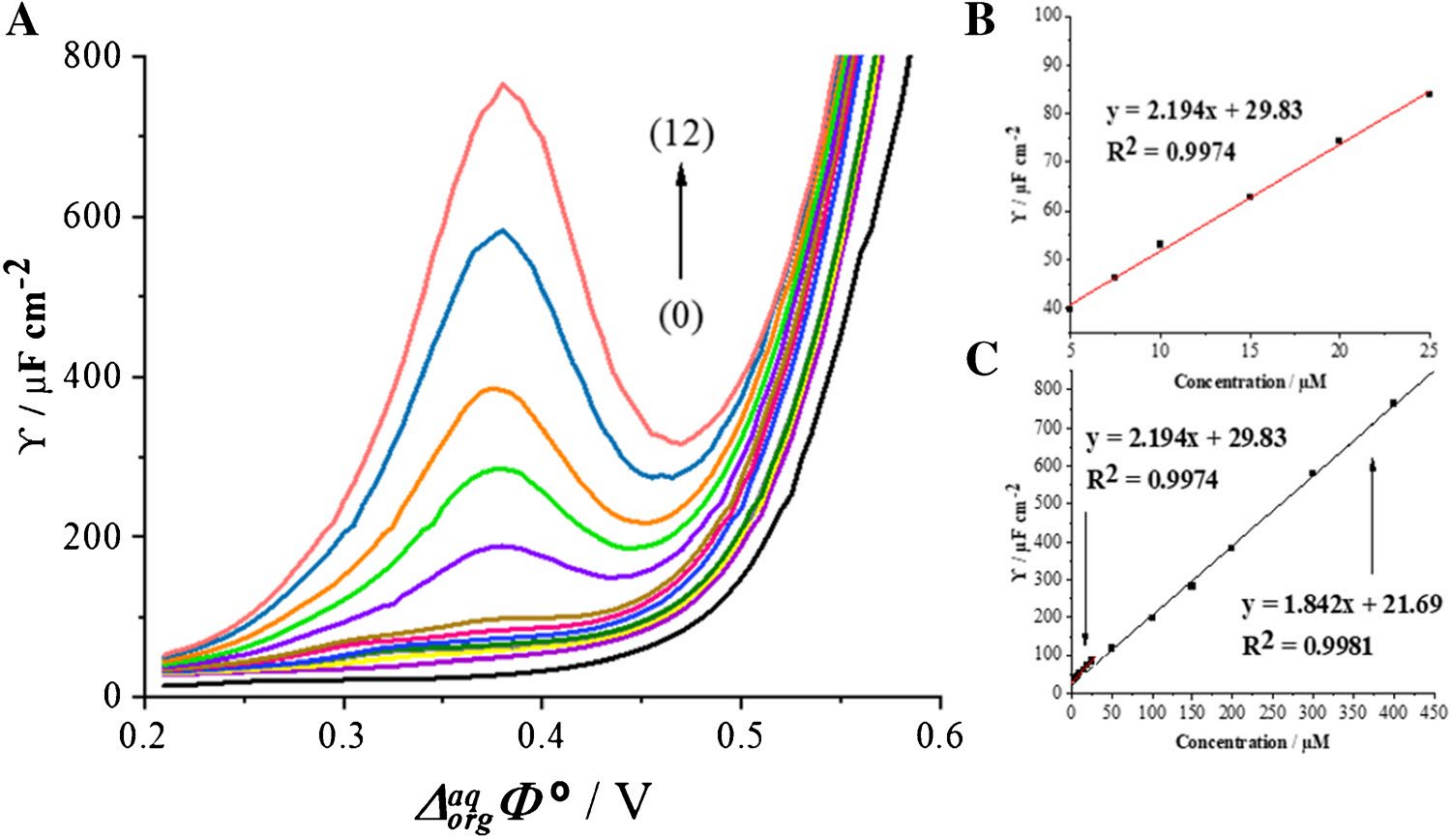
**A** Alternating current voltammograms (ACVs) recorded for increasing concentration of CTX^+^. Two dynamic linear ranges (LDRs) are recorded: (0) blank, LDR_1_: (1) 5.00; (2) 7.50; (3) 10.00; (4) 15.00; (5) 20.00; (6) 25.00; LDR_2_: (6) 25.00; (7) 50.00; (8) 100.0; (9) 150.0; (10) 200.0; (11) 300.0; (12) 400.0 μM. **B** The inset presents the corresponding calibration curve for the LDR_1_. **C** The corresponding signal intensities plotted in a function of the CTX^+^ concentration. Conditions: *f* = 1 Hz, *E* = 10 mV, and Δ*E* = 10 mV.

Recently, Suárez-Herrera and Scanlon [34] applied ACV technique to quantitatively analyze simple ion transfer reactions across the electrified LLI. Measurements in AC voltammetry technique are based on the registration of impedance spectra and then converting them to analytical signals. The employment of electrochemical impedance spectroscopy (EIS) allows for the complete elimination of the capacitive current and thus a significant improvement of the sensitivity of the applied technique. In this work, we have applied the optimized ACV procedure for the determination of CTX^+^ at the macroITIES. Figure 2A shows the ACV curves recorded at the polarized 10 mM HCl || 1,2-DCE interface in the conventional macroITIES cell (Scheme 1) under conditions described in the “Apparatus” section. Figure 2B and C represent the calibration curve exhibiting two linear dynamic ranges, first from 5.00 to 25.00 μM and second from 25.00 to 400.0 μM. The existence of two slopes defined by a signal originating only from a simple ion transfer reaction is unlikely, and hence, we deduced that CTX^+^ may undergo weak adsorption process to the ITIES leading to its interfacial preconcentration. The LOD (1.19 μM) and LOQ (3.98 μM) values for CTX^+^ were calculated from the calibration curve liner fit parameters using *LOD* = 3 × SD_b_/a and *LOQ* = 10 × SD_b_/a (*SD*, the standard deviation; *a*, slope; *b*, intercept), respectively. The values of sensitivities of the CTX^+^ detection using ACV procedure were taken from the calibration curves and are defined as slopes of the linear fitting. All determined analytical parameters are collected in Table 2.

**Table 2.** Electroanalytical parameters of CTX^+^ obtained at the macro- and μITIES.

ITIES dimensionality	macroITIES		μITIES
Technique	CV	ACV	CV
Number of repetitions	3	1	3
LDR [μM]	21.4–909.1	I 5.00–25.00 II 25.00–400.0	13.4–476.2
Slope (*a*) (A M^−1^) for CV–macroITIES (F cm^−2^ M^−1^) for ACV–macroITIES (nA μM^−1^) for μITIES	0.14 ^aq→org^ − 0.12 ^org→aq^	I 2.19 II 1.84	0.003 ^aq→org^ − 0.004 ^org→aq^
Standard error of slope (*SE*_*a*_) ^[a]^	0.00 ^aq→org^ 0.00 ^org→aq^	I 0.06 II 0.04	0.000 ^aq→org^ 0.000 ^org→aq^
Intercept (*b*) (μA) for CV–macroITIES (μF cm^−2^) for ACV–macroITIES (nA) for μITIES	4.852 ^aq→org^ − 0.805 ^org→aq^	I 29.83 II 21.69	0.068 ^aq→org^ − 0.094 ^org→aq^
Standard error of intercept (*SE*_*b*_) ^[a]^	0.568 ^aq→org^ 0.970 ^org→aq^	I 0.872 II 7.723	0.025 ^aq→org^ 0.022 ^org→aq^
Coefficient of determination (*R*^*2*^)	0.9993 ^aq→org^ 0.9970 ^org→aq^	I 0.9974 II 0.9981	0.9939 ^aq→org^ 0.9965 ^org→aq^
LOD (μM) ^[b]^	3.53 ^aq→org^ 2.71 ^org→aq^	1.19	1.46 ^aq→org^ 2.22 ^org→aq^
LOQ (μM) ^[c]^	11.76 ^aq→org^ 9.03 ^org→aq^	3.98	4.86 ^aq→org^ 7.39 ^org→aq^

^[a]^*SE = SD/n*^*1/2*^; ^[b]^*LOD* = 3SD_b_ / a; ^[c]^*LOQ* = 10SD_b_ / a.

a, slope; and b, intercept

### CTX^+^ detection at μITIES

Modern analytical procedures should obey the principles defined by the green chemistry philosophy. One of the trends we should all follow is to minimize the consumption of chemicals, especially those that are toxic. The miniaturization of electrochemical systems based on ITIES undoubtedly meets these expectations. Moreover, ITIES downscaling meeting specific geometrical recruitments brings a few additional benefits to electroanalytical sensing: (I) stabilization of the electrified LLI by the employed support (capillary forces and surface wettability of the used supports define the position of the soft junction); (II) improved detection sensitivity of the voltammetric methods (shape of the diffusion zones established above miniaturized LLI enhances the mass transport from the bulk solution to the polarizable junction); and (III) better analytical performance in terms of LOD and LOQ (reduction of the electroactive surface area = smaller capacitive current) [27, 35]. Therefore, in this work, we have used the FSTM as a polarized LLI support and further applied resulting platform in CTX^+^ detection.

**Fig. 3.**
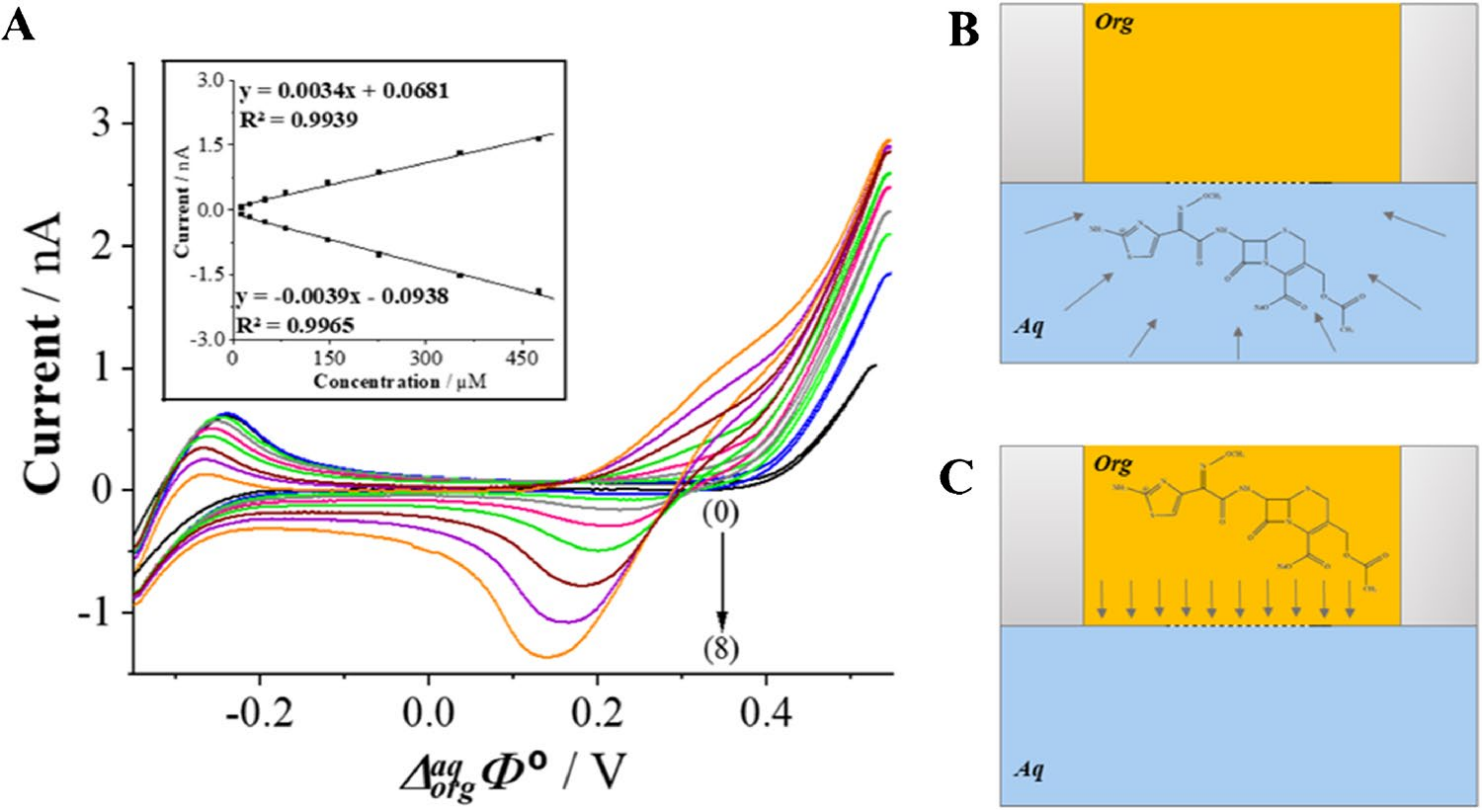
**A** Representative CVs recorded for increasing CTX^+^ concentrations of (1) 13.3; (2) 26.6; (3) 49.8; (4) 82.6; (5) 147.8; (6) 228.0; (7) 353.7; (8) 476.2 μM; and blank (0) in 10 mM HCl (*pH* = 2) used as the aqueous phase. The inset presents forward and backward *I*_*s*_ intensities plotted in a function of increasing *c*_*CTX+*_. **B** The scheme of interfacial mass transfer of CTX^+^ controlled by the hemispherical diffusion. **C** The backward CTX^+^ transfer occurring inside the FSTM governed by a linear diffusion. Conditions: *v* = 20 mV s^−1^.

The μ-devices after preparation and characterization were employed to investigate the interfacial behavior of CTX^+^ in 10 mM HCl solution (*pH* = 2). To evaluate the utility of CV procedure for CTX^+^ determination at the polarized μITIES, CTX^+^ concentration (*c*_*CTX+*_) dependency was recorded (Fig. 3A), and the corresponding calibration curves of *I*_*s*_ *vs. c*_*CTX+*_ were plotted (the inset of Fig. 3A). The asymmetric shape of recorded voltammograms is caused by the coexistence of a hemispherical (CTX^+^ transfer from the aqueous to the organic phase, backward scan, Fig. 3B) and linear diffusion (CTX^+^ transfer from the organic to the aqueous phase, forward scan, Fig. 3C) and on each side of the LLI. Such behavior is described in other published works [14, 27]. The signal limiting the potential window on the less positive potential window side (~− 0.25 V) originate from the interfacial transfer of Cl^-^ anions or BTPPA^+^ cations (Cl^-^ transfer from the aqueous to the organic phase or BTTPA^+^ from the organic to the aqueous phase is expected to be recorded as the negative current). Calibration curve was linear within the entire studied concentration range, this is from 13.32 to 476.2 μM, for both the positive and negative signal intensities. The calculated LOD values for the forward and backward signal currents are equal to 1.5 and 2.2 μM, respectively. Remaining determined analytical parameters, LOQs, and sensitivities are summarized in Table 2.

### Real sample analysis

**Fig. 4.**
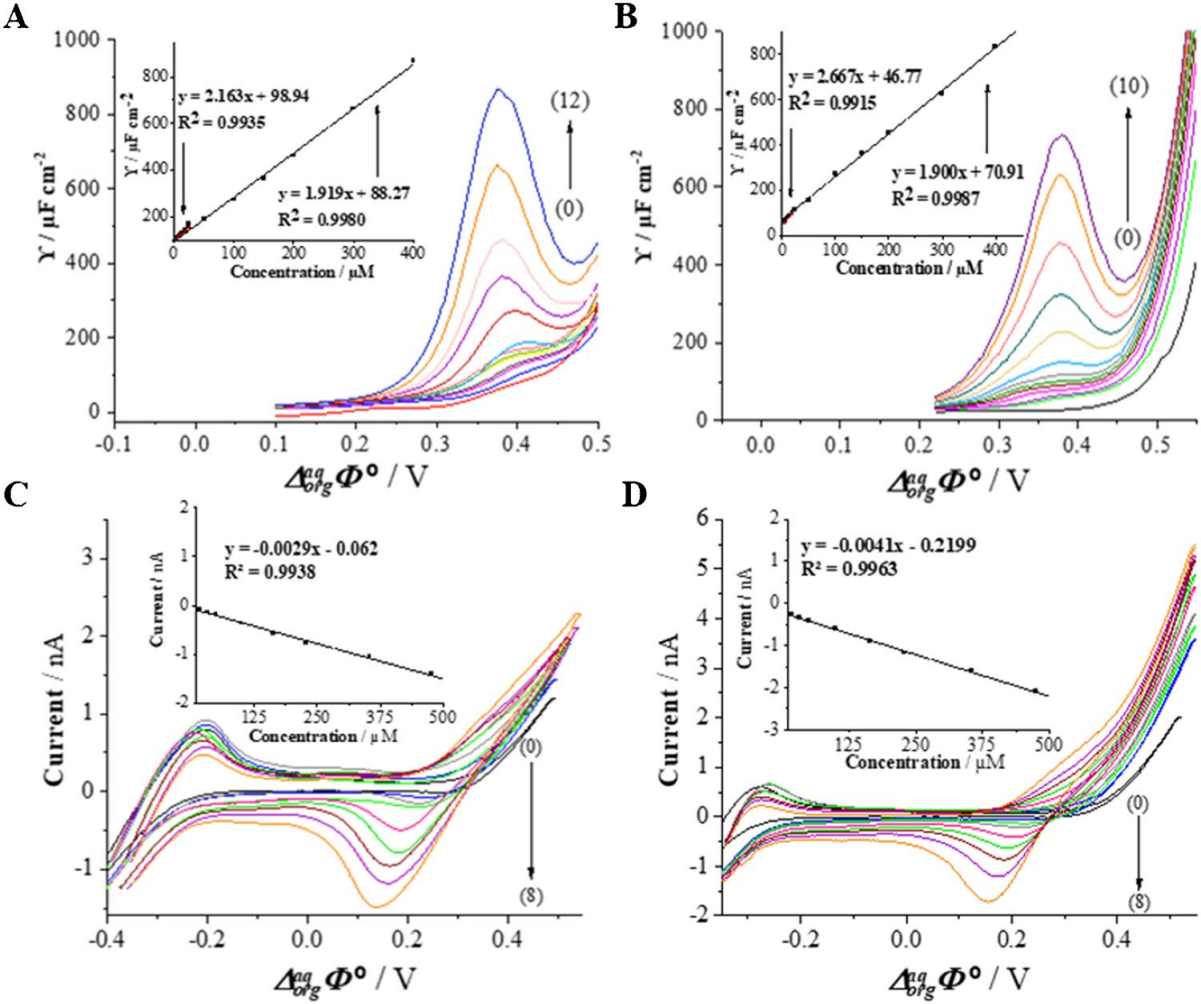
**A**, **B** ACVs recorded at macroITIES for increasing CTX^+^ concentrations added to still mineral (**A**) and tap water (**B**) together with the corresponding calibration curves showing the intensities of the signal plotted in function of the increasing CTX^+^ concentrations within the range of (1) 5.00; (2) 7.50; (3) 10.00; (4) 15.00; (5) 20.00; (6) 25.00; (7) 50.00; (8) 100.0; (9) 150.0; (10) 200.0; (11) 300.0; (12) 400.0 μM; and blank (0). Conditions ACV: *f* = 1 Hz, *E* = 10 mV and Δ*E* = 10 mV, *pH* = 2. **C**, **D** CVs recorded at μITIES for increasing CTX^+^ concentrations added to still mineral (**C**) and tap water (**D**) together with the corresponding calibration curves showing the intensities of the backward (negative) currents plotted in function of the increasing CTX^+^ concentrations within the range of (1) 16.64; (2) 33.22; (3) 49.75; (4) 99.01; (5) 163.9; (6) 228.0; (7) 322.58; (8) 476.2 μM; and blank (0). Conditions CV: scan rate = 20 mV·s^−1^, pH = 2.

To verify the applicability of the developed method, CTX^+^ was determined in a spiked tap and mineral water samples using CV and ACV techniques (Fig. 4). The pH of collected water samples was first adjusted with a 1 M HCl solution until *pH* = 2 was reached. Next, appropriate aliquots of CTX^+^ stock solutions were added. The samples did not require any further purification to remove salts or any other contaminants. The chemical composition of real tap and mineral water samples includes inorganic cations that may undergo interfacial ion transfer further affecting the positive current values limiting the potential window on more positive potential site. We assume that the existence of additional cations in the aqueous phase is a consequence of overlaid positive current signals recorded for real samples shown in Fig. 4C/D. As such, the backward signals were analyzed for CTX^+^ quantification.

### AC voltammetry measurements

The ACV studies were performed in a traditional macroITIES cell (Scheme 1), wherein the aqueous phase compartment of the cell was filled with 3.50 mL of a sample (mineralor tap water). Figure 4 presents the ACVs recorded upon addition of the appropriate aliquot of the stock solution of CTX^+^ to the still mineral water (Fig. 4A) and tap water (Fig. 4B) samples. The analytical signals (positive or negative currents) increased linearly with CTX^+^ concentration in two linear dynamic ranges of 5.00 to 25.00 μM and 25.00 to 400.0 μM in both samples. The LODs and LOQs were calculated from the calibration curves and first LDR. The calculated LODs values for determination of CTX^+^ in a mineral and tap water samples are equal to 1.9 and 2.1 μM, respectively (the lowest detected concentration was equal to 1.00 μM and was beyond the LDR). Given obtained output, described procedure is suitable for CTX^+^ determination in real samples.

### μITIES measurements

The utility of the elaborated CV procedure at the μITIES was also checked in determination of CTX^+^ in a still mineral (Fig. 4C) and tap (Fig. 4D) water samples. The CV measurements were conducted at μITIES platform (Scheme 2) immersed to the aqueous phase having a total volume of 15.00 mL (mineral or tap water). The calibration curves were constructed only based on the backward (negative) peak current because the signals of CTX^+^ transfer from the aqueous to the organic phase were overlapped with the proton transfer. As can be seen from the Fig. 4, the corresponding calibration curve is linear within the dynamic range of 16.6–476.2 μM CTX^+^ concentrations in both samples. The LODs values for CTX^+^ determination in a tap and mineral water samples are calculated to be 5.57 and 9.70 μM, respectively. All obtained analytical parameters for CTX^+^ determination in real samples by means of ACV and CV techniques were collected in Table S1 (see the electronic supporting information).

## Conclusion

This work reports on quantitative analysis performed by two electrochemical techniques: cyclic voltammetry (CV) and alternating current voltammetry (ACV), for the determination of the antibiotic — cefotaxime in model — still mineral and tap water samples. The output of the ACV as compared with CV method applied to a macroITIES provided better analytical parameters (lower LODs and LOQs values). Although giving better performance, ACV technique requires further data processing and is more time-consuming. The application of the μITIES-based setup for the real sample analysis additionally provided improved stability of the soft junction supported with a solid support, reduced volume of the organic phase, and assured compact size of the sensing set-up. Cefotaxime detection at μITIES with CV provided improved electroanalytical parameters comparable to these obtained with ACV at macroITIES. Hence, miniaturized systems were further applied for cefotaxime detection in real samples. Since we did not observe any interference of the real sample matrix during cefotaxime detection, these did not require any further preparation.

## Supplementary information

ESM 1 (DOCX 798 kb)
