# Supplementary material for: Voltammetric study of cefotaxime at the macroscopic and miniaturized interface between two immiscible electrolyte solutions
Source: Mikrochim Acta. 2021 Nov 9;188(12):413. doi: 10.1007/s00604-021-05072-w (PMC8578136; doi:10.1007/s00604-021-05072-w)
Supplement: Supplementary file 1 — (DOCX 798 kb) [file 604_2021_5072_MOESM1_ESM.docx]

**Electronic Supplementary Material**

**Voltammetric study of cefotaxime at the macroscopic and miniaturized interface between two immiscible electrolyte solutions**

**Konrad Rudnicki,^a^* Karolina Sobczak,^a^ Magdalena Kaliszczak,^b^ Karolina Sipa,^a^ Emilia Powałka,^a^ Sławomira Skrzypek,^a^ Lukasz Poltorak,^a**^ Gregoire Herzog,^b***^**

^a^Department of Inorganic and Analytical Chemistry, Faculty of Chemistry, University of Lodz, Tamka 12, 91-403 Lodz, Poland

^b^Université de Lorraine, CNRS, LCPME, Nancy, France

*Corresponding author: konrad.rudnicki@chemia.uni.lodz.pl

**Corresponding author: lukasz.poltorak@chemia.uni.lodz.pl

***Corresponding author: gregoire.herzog@univ-lorraine.fr





**Fig. S1.** Concentration fraction of all forms of CTX plotted in a function of the pH of the aqueous phase.  *pK_a1_* and *pK_a2_* are marked with black dashed lines perpendicularly cutting the pH axis at their values. Existing CTX forms are marked in the figure legend.


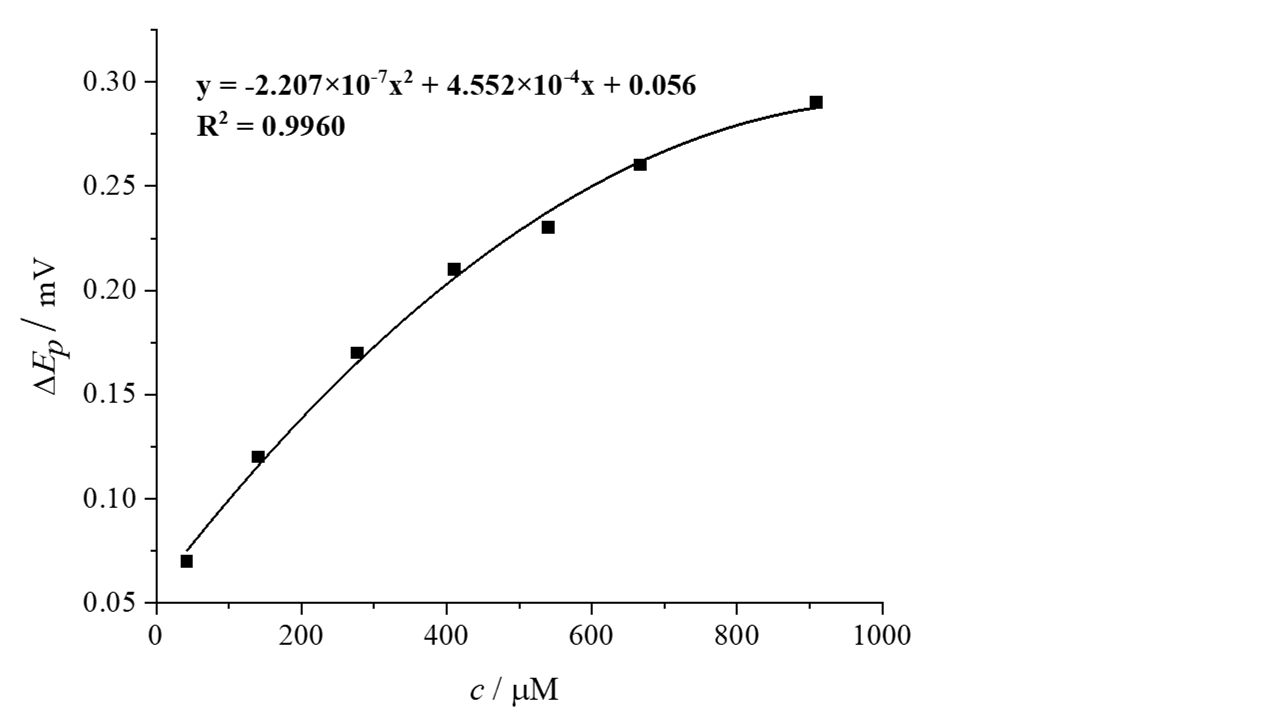


**Fig. S2.** The plot of the peak-to-peak separation (Δ*E_p_*) *vs.* CTX concentration (*c*) within the range 21.38 – 909.1 µM.


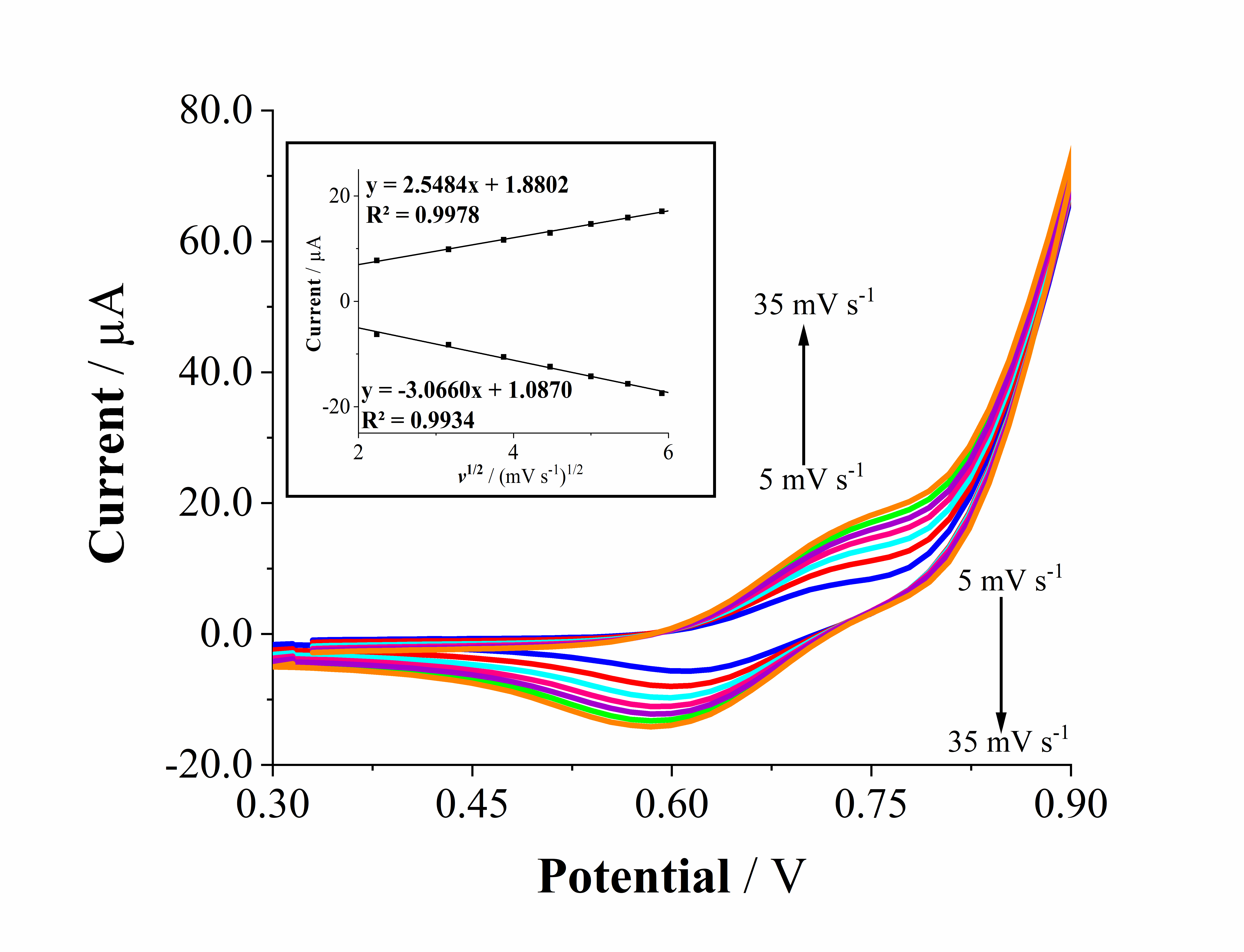


**Fig. S3.** Scan rate dependency for the [CTX] = 140.8 μM recorded at macroITIES for increasing scan rates: 5; 10; 15; 20; 25; 30 and 35 mV s^-1^, aqueous phase was 10 mM HCl, pH = 2. The inset shows corresponding plots representing the dependency between forward (positive) and backward (negative) current signals plotted in a function of the square root (*v^1/2^*) of the scan rate.

**Characterization of µITIES systems**

Prior to electroanalytical experiments, each constructed FSMT based sensing platform was analyzed using SEM. Visual inspection allowed the evaluation of the pore diameter and its comparison with the value declared by manufacturer (25 µm). Next, FSMT based platforms were investigated using ITV in the presence of tetramethylammonium cation (TMA^+^) at fixed concentration dissolved in the aqueous phase (see Fig. S4-A). Based on the value of the steady state current attributed to the TMA^+^ transfer from the aqueous to the organic phase (positive current) and Saito equation (eq. 1) we have calculated the internal diameter of the FSMT.

$I_{ss}=4zDcFr$ (Eq. 1)

where: *I_ss_* - the steady-state current (31 nA taken from Fig. S4-A); *z* - the charge of the model ion (z = 1) *D* - the water phase diffusion coefficient for TMA^+^ (13.8×10^−6^cm^2^ s^−1^) [1]; *c* – TMA^+^ concentration (50 µM); and *r* is the radius of the µ-aperture. Using the *I_ss_* value (0.31 nA) extracted from voltammogram recorded in the presence of TMA^+^ (Fig. S4-A) we have calculated the µITIES internal diameter equal to 23.2 µm, which is reasonably close to the 24.15 µm deduced from SEM micrography (see Fig. S4-B). The values of the µ-aperture diameter obtained experimentally either with ITV or SEM are comparable and the difference between them not exceed 5%.


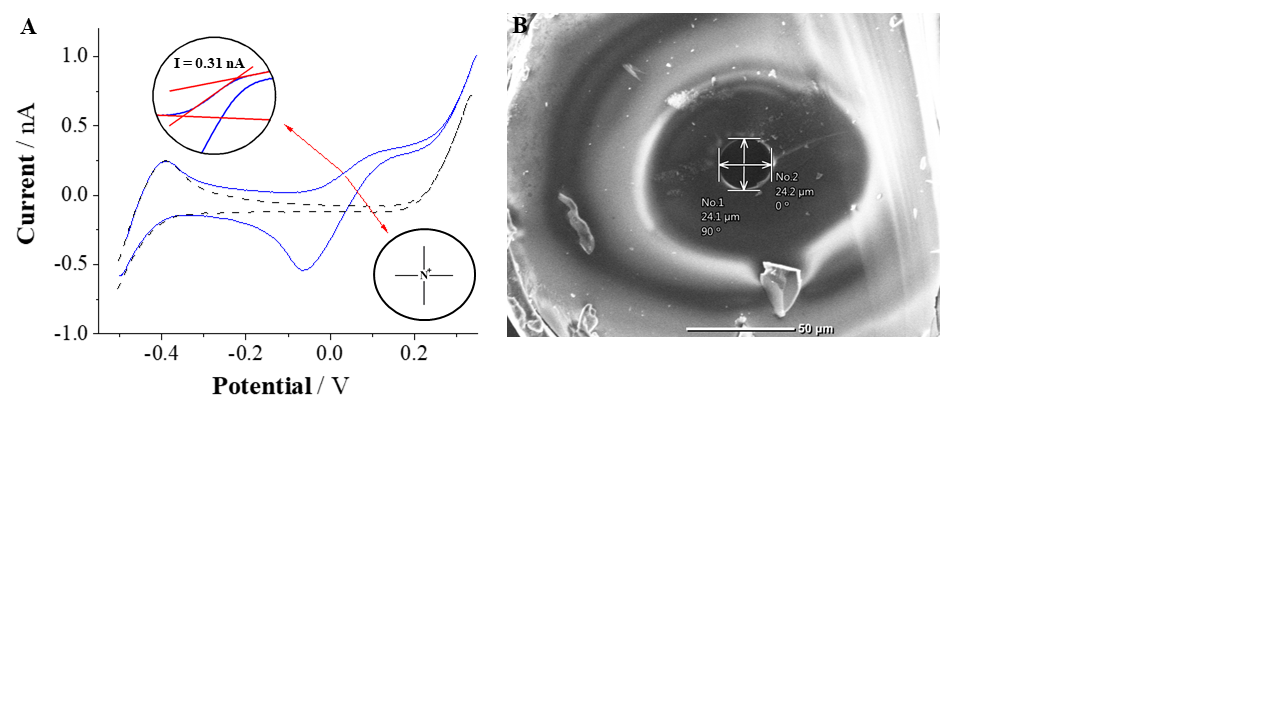


**Fig. S4.** A – Selected ITVs recorded in the presence of [TMA^+^] = 50 μM (solid, blue line) and blank (dashed, black line) recorded at µITIES supported with a fused silica capillary. Conditions: 10 mM HCl, pH = 2; *v* = 20 mV s^-1^. B – SEM micrographs of FSMT after a cut with a ceramic knife.

**Table S1**. Electroanalytical parameters obtained for CTX determination in real samples by means of ACV and ITV technique.

| **Type of sample** | Soda water | Tap water | Soda water | Tap water |
| --- | --- | --- | --- | --- |
| **Technique** | ACV | | ITV | |
| **LDR [μM]** | I 5.00 – 25.00  II 25.00 – 400.0 | | 16.64 – 476.2 | |
| **Slope (*a*)**  **(F cm^-2^ M^−1^) for ACV**  **(nA µM^−1^) for µITIES** | I 2.163  II 1.919 | I 2.667  II 1.907 | -0.003 | -0.004 |
| **Standard error of slope (*SE_a_*) ^[a]^** | I 0.088  II 0.038 | I 0.123  II 0.030 | 0.000 | 0.000 |
| **Intercept (*b*) (μF cm^-2^)**  **(nA) for µITIES** | I 98.94  II 88.27 | I 46.77  II 70.91 | -0.062 | -0.220 |
| **Standard error of intercept (*SE_b_*) ^[a]^** | I 1.35  II 8.28 | I 1.91  II 6.55 | 0.022 | 0.023 |
| **Coefficient of determination (*R^2^*)** | I 0.9935  II 0.9980 | I 0.9915  II 0.9987 | 0.9938 | 0.9963 |
| **LOD (μM) ^[b]^** | 1.88 | 2.14 | 5.57 | 9.70 |
| **LOQ (μM) ^[c]^** | 6.26 | 7.15 | 18.56 | 32.34 |

^[a]^ *SE = SD/n^1/2^*; ^[b]^ LOD = 3SD_b_ / a; ^[c]^ LOQ = 10SD_b_ / a.
a – slope and b – intercept

***References***

1. Hinoue T, Ikeda E, Watariguchi S, Kibune Y (2007) Thermal modulation voltammetry with laser heating at an aqueous|nitrobenzene solution microinterface: Determination of the standard entropy changes of transfer for tetraalkylammonium ions. Anal Chem 79:291–298. https://doi.org/10.1021/ac061315l
